# Viral Suppressors of RNA Silencing Hinder Exogenous and Endogenous Small RNA Pathways in *Drosophila*

**DOI:** 10.1371/journal.pone.0005866

**Published:** 2009-06-10

**Authors:** Bassam Berry, Safia Deddouche, Doris Kirschner, Jean-Luc Imler, Christophe Antoniewski

**Affiliations:** 1 Institut Pasteur, Drosophila Genetics and Epigenetics, CNRS-URA2578, Paris, France; 2 IBMC, CNRS-UPR9022, Strasbourg, France; Victor Chang Cardiac Research Institute (VCCRI), Australia

## Abstract

**Background:**

In plants and insects, RNA interference (RNAi) is the main responder against viruses and shapes the basis of antiviral immunity. Viruses counter this defense by expressing viral suppressors of RNAi (VSRs). While VSRs in *Drosophila melanogaster* were shown to inhibit RNAi through different modes of action, whether they act on other silencing pathways remained unexplored.

**Methodology/Principal Findings:**

Here we show that expression of various plant and insect VSRs in transgenic flies does not perturb the *Drosophila* microRNA (miRNA) pathway; but in contrast, inhibits antiviral RNAi and the RNA silencing response triggered by inverted repeat transcripts, and injection of dsRNA or siRNA. Strikingly, these VSRs also suppressed transposon silencing by endogenous siRNAs (endo-siRNAs).

**Conclusions/Significance:**

Our findings identify VSRs as tools to unravel small RNA pathways in insects and suggest a cosuppression of antiviral RNAi and endo-siRNA silencing by viruses during fly infections.

## Introduction

RNA silencing is a eukaryotic gene regulation mechanism by which RNA expression is shut down in a sequence specific manner through the intervention of homologous small non coding RNAs [1]. Three types of small RNAs, miRNAs, piRNAs and siRNAs, accumulate in different tissues and developmental stages of *Drosophila*.

The ∼22 nucleotides (nt) miRNAs derive from stem-loop precursor transcripts through the action of the Drosha/Pasha and Dicer-1/Loqs complexes. In *D. melanogaster*, they are mostly loaded into the Argonaute-1 (Ago1) protein and guide translational repression of mRNAs [2], [3]. The ∼25-nt piRNAs are produced from the cleavage of selfish and repetitive genetic element transcripts by the Piwi Argonautes. They are restricted to the gonads where they silence Transposable Elements (TEs) [4]. The ∼21 nt siRNAs originate from the processing by Dicer-2 (Dcr2) of long dsRNA precursors, such as those produced by inverted-repeat (IR) transgenes. They load into Argonaute-2 (Ago2) and guide the cleavage of target mRNAs with perfect complementary sequence matches [5]. siRNAs produced from endogenous precursors (endo-siRNAs) were recently described in flies. Beside piRNAs, they contribute to the silencing of TEs and some endogenes in both gonads and somatic tissues [1], [4], [6].

siRNAs are also produced by Dcr-2 from dsRNA viral intermediates upon viral infection [7]–[9], playing an essential role in the fly's defense against viruses. To counterattack, viruses evolved to encode proteins that suppress their host's antiviral silencing. Viral suppressors of RNAi (VSRs) have been reported in all types of plant viruses [10]. More recently, insect viral suppressors, the *Flock House virus* (FHV) B2, the *Cricket Paralysis virus* (CrPV) and the *Drosophila C virus* (DCV) 1A proteins where shown to act as potent inhibitors of antiviral RNAi in Drosophila [8], [9], [11]. VSRs are very diverse in sequence and structure across viral kingdoms, but operate through a few evolutionarily convergent strategies: they most commonly bind to long dsRNAs to inhibit their processing by Dicer proteins [12]–[14]; sequester siRNA duplexes to prevent their loading to Argonaute complexes [15]–[17]; or may directly interact with Dicers or Argonautes to impair their antiviral activities [18], [19].

Alongside inhibiting antiviral RNAi, plant VSRs including HcPro, P21, P19, P15 and P0 were shown to suppress miRNA- or endo-siRNA-mediated silencing, therefore corrupting development or homeostasis of their host and contributing to viral pathogenicity [18], [20]–[23]. In contrast, the effect of VSRs on endo-siRNAs in animals and its potential contribution to host disorders has not been explored in detail. To address this issue, we established *Drosophila* transgenic lines expressing a panel of 9 VSRs from plant and insect viruses (Table 1) and performed a comparative analysis of their effects on RNAi induced by (i) viral infection, (ii) inverted repeat transgenes, (iii) *in vitro* transcribed dsRNA, (iv) synthetic siRNAs, (v) endogenous siRNAs and (vi) miRNAs.

**Table 1:** Viral Suppressors of RNAi used in this study.

Host	VSR	Virus	Genus	Mode of action
Insects	B2	*Flock house virus*	Nodavirus	dsRNA and siRNA binding [12], [14], [26], [27]
	1A	*Drosophila C virus*	Cripavirus	long dsRNAs binding [8]
Plants	P0	*Cucurbit aphid born yellows virus*	Luteovirus	ubiquitilation degradation of Argonautes proteins [38]
	P15	*Peanut clump virus*	Pecluvirus	siRNA binding [35]
	P19	*Tomato bushy stunt virus*	Tombusvirus	siRNA binding [15], [16]
	P21	*Beet yellows virus*	Closterovirus	siRNA binding [35]
	P25	*Potato virus X*	Potexvirus	unknown [21], [42]
	P38	*Turnip crinkle virus*	Carmovirus	interferes with DCL4 [43]
	HcPro	*Zucchini yellow mosaic virus*	Potyvirus	siRNA binding [17]

Our study reveals that none of these VSRs perturb the miRNA pathway. In contrast, the insect VSRs FHV B2 and DCV 1A, as well as the plant VSRs P21, P19 and P15, inhibited anti-viral RNAi in flies and suppressed silencing induced by siRNAs either exogenously supplied or endogenously produced, strongly supporting the relevance of RNAi mechanisms throughout evolution and kingdoms. In addition, B2, 1A and P19 suppressed the silencing of TEs in somatic tissues and in gonads, suggesting that effects of VSRs on endo-siRNA-mediated silencing may account for viral pathogenicity in insects. Our results demonstrate the effectiveness of using viral silencing suppressor proteins as a tool to dissect small RNA pathways in animals, as previously established in plants.

## Results

### VSRs do not inhibit the Drosophila miRNA pathway

*Drosophila* transgenic lines were established to express a panel of 9 Flag-tagged VSRs (Table 1) under the control of a GAL4 inducible UAS promoter [24]. Two VSRs, B2 and 1A, derive from the insect viruses FHV and DCV, respectively. The remaining VSRs P0, HcPro, P25, P38, P21, P15 and P19 derive from plant viruses. The VSR transgenic lines were crossed with a *daughterless*-GAL4 driver line, which ubiquitously expresses GAL4 throughout development. Western blot assay using a Flag antibody indicated that all suppressors are expressed at a high level in the progenies from these crosses (Fig. S1).

In plants, HcPro, P21, P19, P15 and P14 interfere with miRNAs, causing developmental defects that resemble those observed in mutant plants deficient in their miRNA pathway [20]–[23]. In addition, it has been reported that the P19 VSR might interfere with the silencing activity of miR-32 in human cells [25]. Thus, we assessed the effect of the overexpression of the 9 VSRs on miRNA activity. Expression of the VSRs in the posterior compartment of the wing imaginal discs did not suppress the silencing of a GFP reporter targeted by the bantam miRNA (Fig. S2 and Movies S1 and S2). Additionally, the transgenic stocks ubiquitously expressing each of the 9 VSRs progressed normally from embryogenesis to adulthood, showing neither developmental defects nor altered viability. These results indicate that the VSRs here tested do not appreciably perturb the miRNA pathway in *Drosophila*.

### 

#### Suppression of antiviral RNAi

**Figure 1:**
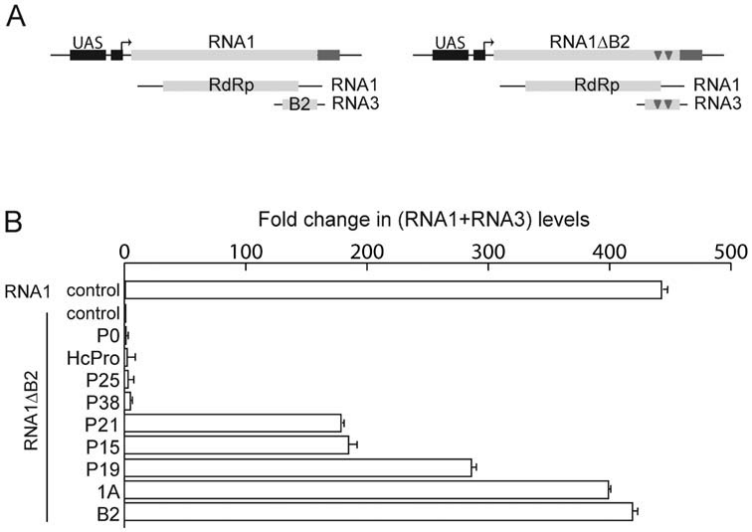
Flies expressing insect and plant VSRs accumulate high levels of FHV RNA1ΔB2 transcripts. (A) RNA1 and RNA1ΔB2 constructs. The GAL4 inducible UAS-RNA1 transgene (left hand side) expresses a RNA1 of (+) polarity. RNA1 encodes an RdRp protein, which directs autonomous replication of RNA1 of (−) polarity, then of both RNA1 and RNA3 of (+) polarity. Two point mutations (triangles) interrupt the B2 ORF in the RNA1ΔB2 (right hand side) without affecting the RdRp ORF [7]. (B) Fold changes in RNA1+RNA3 levels. The RNA1 transgenic line was crossed with the *da*>GAL4 line (control). The RNA1ΔB2 transgenic line was crossed with the *da*>GAL4 line (control) or with *da*>GAL4; UAS-VSR lines as indicated. RNA1+RNA3 expression was measured by quantitative RT-PCR from the progenies of these crosses. Error bars indicate the standard deviation from triplicate qPCR experiments.

A FHV RNA1 transgenic construct was previously engineered to autonomously replicate in the presence of the viral RNA-dependent RNA polymerase (RdRp) encoded by RNA1 [7]. Non-sense mutations in the B2 ORF of RNA1 (Fig. 1A) strongly impair the replication of a derivative RNA1ΔB2 transgenic construct (Fig. 1B, compare RNA1 and RNA1ΔB2 controls), an effect that was attributed to the inability of RNA1ΔB2 to limit the RNAi host response through the activity of the B2 VSR [7]. Accordingly, RNA1ΔB2 expression was restored in flies expressing the B2 transgene (Fig. 1B). We therefore decided to monitor the ability of our panel of VSRs to restore the replication level of RNA1ΔB2 (Fig. 1B).

RNA1ΔB2 replication was restored in DCV 1A transgenic flies at levels similar to those observed in B2 transgenic flies. High levels of RNA1ΔB2 replication were also measured in flies expressing P15, P21 or P19, suggesting that these proteins, in a similar way to B2 and 1A, suppress fly antiviral RNAi. In contrast, no significant increase of RNA1ΔB2 replication was observed in flies expressing P38, P25, HcPro or P0.

**Figure 2:**
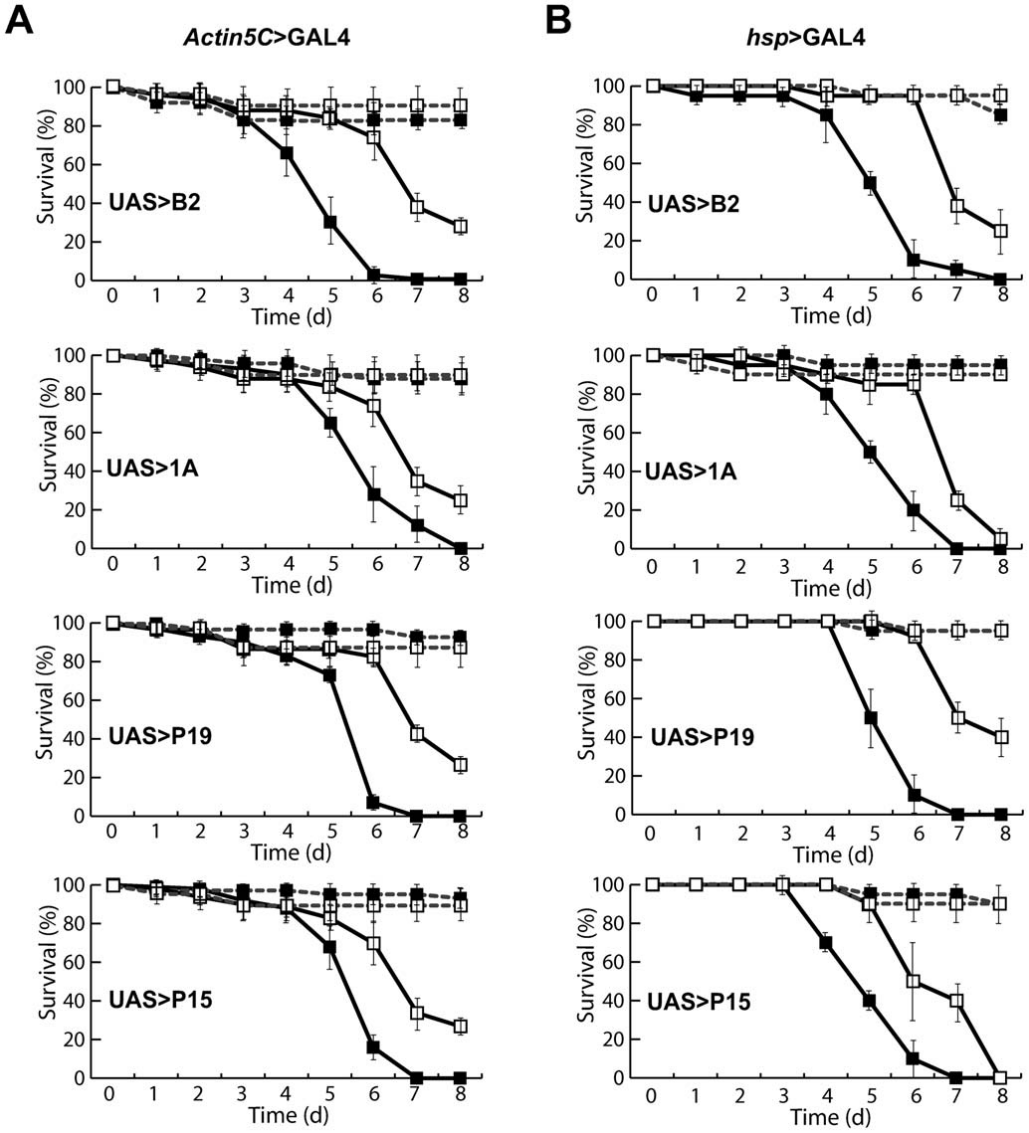
Hypersensitivity to DCV infection. (A) An *Act5C*>GAL4 driver line was crossed to UAS>VSR transgenic lines (▪) or to w1118 control strain (□). 20 females from the progeny of these crosses were challenged by an intrathoracic injection of a control Tris solution (---) or a DCV suspension corresponding to 10^2^ LD50 (–) and survival was monitored daily. (B) An *hsp*>GAL4 driver line was crossed to UAS>VSR transgenic lines. 20 females from the progeny of these crosses were challenged by an injection of the control Tris solution (---) or a the DCV suspension (–) under heat shock (▪) or non heat shock (□) conditions. Plotted values represent the mean±SEM (standard error to the mean) of three independent experiments.

To further characterize the impact of the suppressors on antiviral RNAi, we tested their effect on fly susceptibility to infection by the *Drosophila C virus* (DCV), a common *Drosophila* pathogen. Upon DCV inoculation of wild type flies, 50% of animals died after 6 to 7 days (Fig. 2A). The survival was similar for flies expressing P25, P38, HcPro or P0 under the control of the ubiquitous Actin5C-GAL4 driver (data not shown), paralleling the lack of effect of these proteins on the replication of the FHV RNA1ΔB2. In contrast, flies expressing B2, 1A, P19, P21 and P15 under the control of the ubiquitous Actin5C>GAL4 driver showed a more rapid course of disease and died faster after inoculation with DCV (Fig. 2A and data not shown). Similar results were obtained when these suppressors were expressed under the control of the *heat-shock*>GAL4 driver after heat shock induction (Fig. 2B). These data show that the B2 and 1A insect VSRs, as well as the P19, P15 and P21 plant VSRs act as potent inhibitors of antiviral RNAi in flies.

#### Suppression of silencing induced by exogenous dsRNA

**Figure 3:**
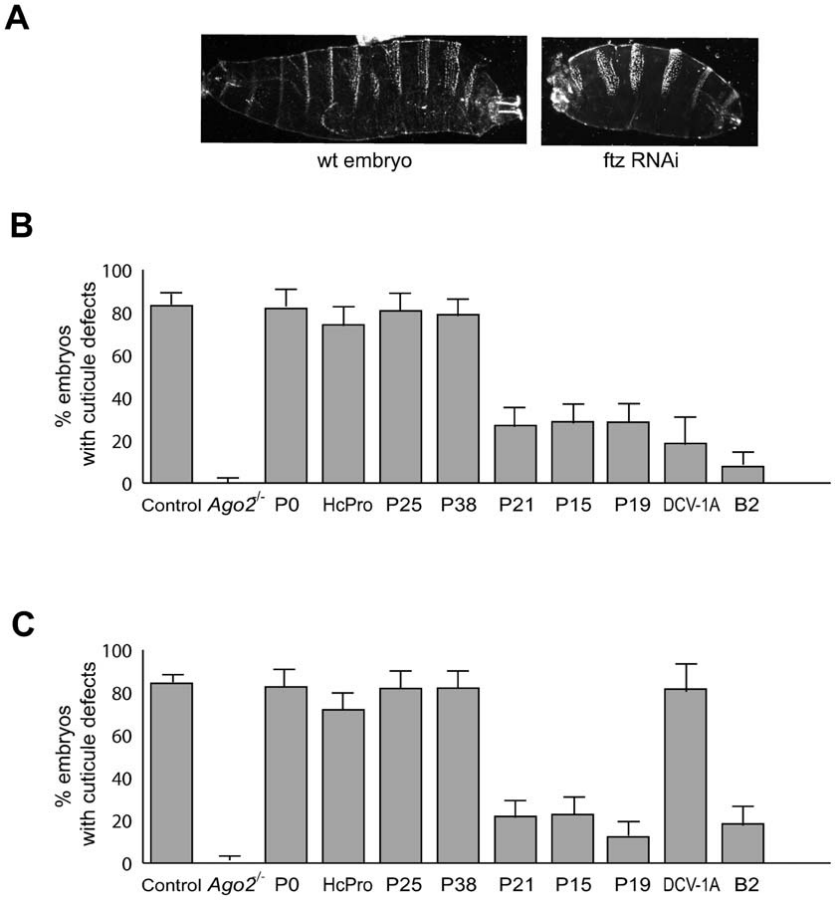
Suppression of silencing induced by injection of long dsRNAs or siRNAs in embryos. RNAi of the *fushi tarazu* gene (*ftz*) in wild type syncytial embryos results in loss of denticle belts in the cuticle of prehatching larvae (A). Early embryos homozygous for the *ago2^414^* mutant allele or expressing the indicated VSR under the control of the *da*>GAL4 driver were injected with long dsRNAs (B) or siRNAs (C) targeted to the *ftz* gene and scored 48 h after injection for the *ftz* mutant phenotype. Confidence intervals (alpha risk 0.05) for the observed ratio are indicated.

Because one of the modes of action of VSR is the binding to long dsRNA species, sequestering them from Dicer access, or to siRNAs hindering their loading into the RISC complex, we next tested the ability of the suppressors to inhibit RNAi induced by injection of dsRNA or siRNA in embryos. 500-nt dsRNAs or 21-nt siRNAs matching the *fushi tarazu* gene (*ftz*) induced a high frequency (>80%) of cuticle defects in wild type survivors, but not in *ago2^−/−^* survivors, indicating that the two types of molecules elicited potent RNAi against the *ftz* gene (Fig. 3). Injection of long dsRNAs or siRNAs in transgenic embryos P0, HcPro, P25 or P38 also induced a strong *ftz* RNAi, with a ∼80% frequency of cuticule defects (Fig. 3B–C). In contrast, only 10–30% of cuticule defects were observed in transgenic embryos P21, P15, P19 or B2, indicating that maternal stocks and/or embryonic expression of these proteins efficiently suppressed *ftz* RNAi. In contrast to B2 transgenic embryos, 1A transgenic embryos were able to mount an efficient *ftz* silencing when injected with short siRNAs but not with long dsRNAs (Compare Fig. 3B and C). This result is fully consistent with previous data indicating that DCV-1A specifically binds long dsRNAs *in vitro* [8], while B2 bind to both long dsRNA [14], [26] and short siRNA [12], [27].

#### Suppression of silencing induced by endogenous siRNAs

**Figure 4:**
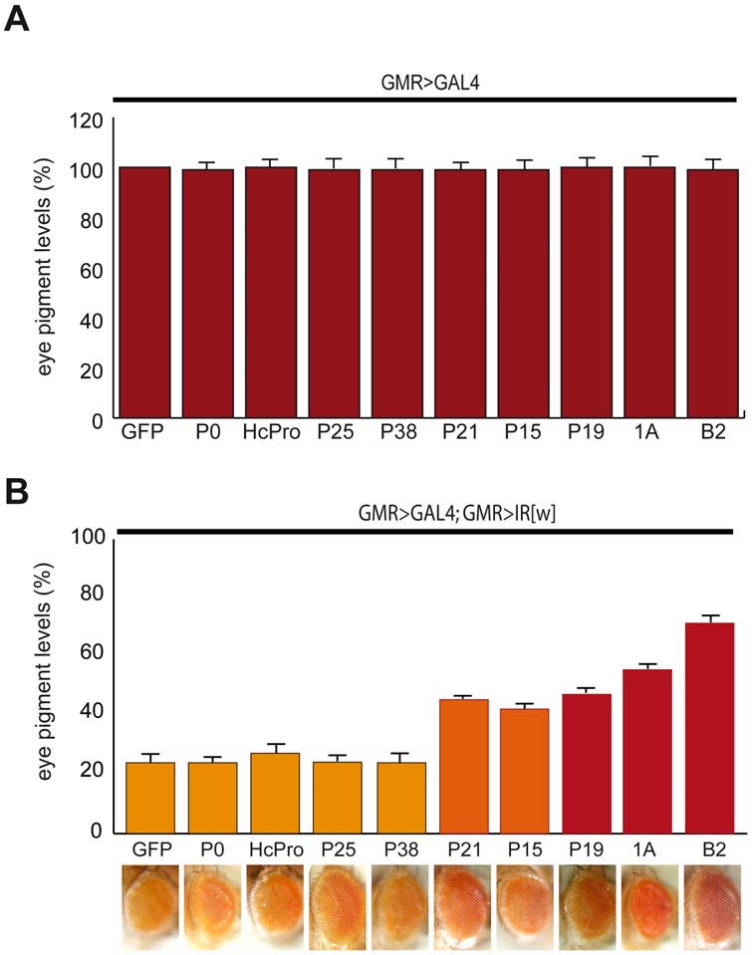
VSRs inhibit silencing induced a *white* inverse-repeat transgene. Silencing of the white gene induced by an IR[w] inverted-repeat transgene in flies expressing insect and plant VSRs in eyes. (A) In all heterozygous combinations *GMR*>GAL4; UAS>GFP or GMR>GAL4; UAS>VSR, the *mini-white* markers of the two transgenes produce equivalent strong red eye pigmentation. (B) Silencing of the *mini-white* markers by one copy of the *GMR*>IR[w] transgene (note that the *GMR*>IR[w] construct has no mini-white marker [28]). The effect of the *GMR*>IR[w] transgene on the two mini-white markers in *GMR*>GAL4; UAS>VSR genetic combinations depends on the efficiency of the suppressor to inhibit *white* silencing. Eye pigment levels were measured in 3 separate experiments from 2 days-old adult eyes and are expressed as a percentage of pigment levels measured in the *GMR*>GAL4; UAS>GFP combination. Error bars correspond to standard deviations.

To establish the role of VSRs on endo-siRNAs we sought to use a *GMR*>IR[w] transgenic construct expressing an RNA hairpin in eyes [28]. This construct triggers the Dcr-2-dependent production of siRNAs [29], [30] and silences the *white* gene (Fig. 4, control panels). When co-expressed under the control of a *GMR*>GAL4 driver with the RNAi trigger *GMR*>IR[w] transgene, B2, 1A and to a lesser extent, P19, P15 and P21, suppressed this *white* silencing (Fig. 4). This result indicates that in addition to antiviral and exogenously induced RNAi, these 5 VSRs can block RNAi induced by an transgenic hairpin locus. In contrast, P38, P25, HcPro and P0 failed to inhibit the *white* silencing, paralleling their lack of effect on antiviral or exogenously induced RNAi.

**Figure 5:**
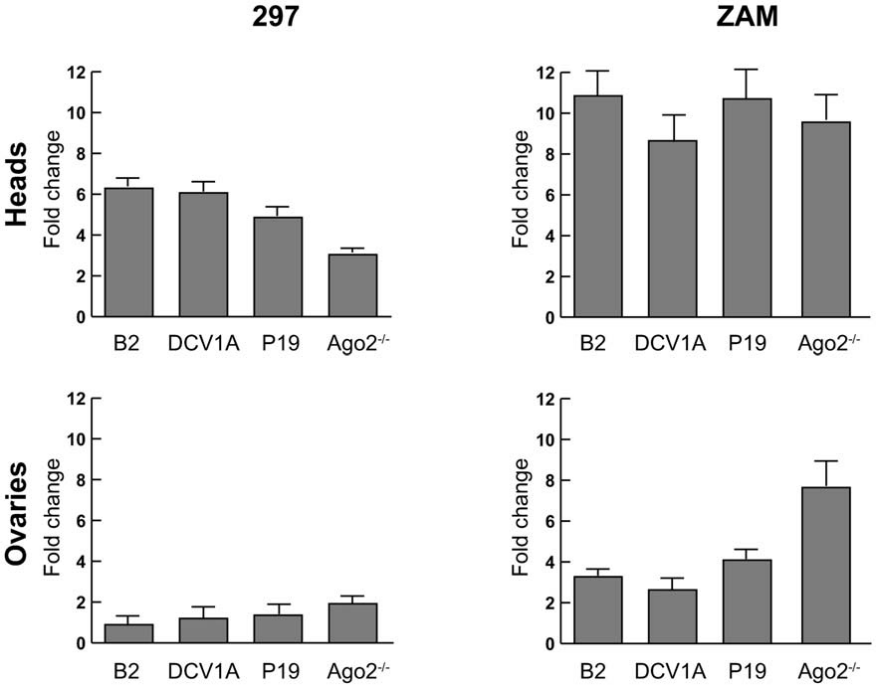
VSRs inhibit retrotransposon silencing by endo-siRNAs. 297 and ZAM retrotransposon silencing is impaired by B2, 1A and P19 VSRs. RNA levels of 297 and ZAM were measured in heads and ovaries of 1 day-old female flies heterozygous for the *da*>GAL4 driver and the indicated UAS>VSR transgene. Fold changes in RNA levels were calculated relative to the 297 and ZAM RNA levels measured in heterozygous *da*>GAL4 control flies. Fold changes in homozygous *ago2^414^* mutants were calculated relative to the 297 and ZAM RNA levels measured in heterozygous *ago2^414^* flies. Therefore, the weaker effect of the *ago2* mutation on 297 expression observed in adult heads may result from variations in copy number of 297 between the *ago2* mutant stock and the VSR transgenic stocks. Error bars indicate the standard deviation from triplicate qPCR experiments.

A class of endogenous siRNAs is produced from sense and antisense transcription of transposable elements. In somatic tissues, these endo-siRNAs were shown to silence TEs from where they originate in a negative feedback loop [4], [6]. To further characterize the activity of B2, 1A and P19 on the fly endo-siRNA pathway, we analyzed the effects of their overexpression on two retrotransposons in female adult heads and ovaries (Fig. 5).

As expected, the steady-state levels of the LTR retrotransposons 297 and ZAM increased in heads from *ago2^−/−^* mutant control flies. The *ago2* mutation also derepressed ZAM and to a lesser extent, 297, in ovaries, agreeing with previous expression level analyses in this tissue [31], [32].

Expression of ZAM increased in heads and to a lesser extent, in ovaries, from B2, 1A and P19 transgenic animals driven by the ubiquitous *da*>GAL4 driver. Expression of 297 also increased in heads but not in ovaries of all three transgenic lines. These data indicate that B2, 1A and P19 impair transposon silencing by endo-siRNAs in somatic tissues. The modest effect of these VSRs in ovaries suggests that they do not interfere with piRNA silencing in this tissue.

## Discussion

Here we analyzed the ability of 9 VSRs to interfere with small RNA silencing pathways in adult flies. We found that 2 insect VSRs - B2 and 1A - and 3 plant VSRs - P19, P21 and P15 - inhibit *in vivo* antiviral RNAi as well as RNAi induced by both exogenously and endogenously supplied dsRNAs.

None of the VSRs tested in this study significantly interfered with miRNA activity. Biogenesis of miRNAs involves primary miRNA processing by Drosha/Pasha in the nucleus, exportin-5 dependent export of pre-miRNAs in the cytoplasm, pre-miRNA processing by Dicer-1 and miRNA loading into RISC complexes. It is possible that VSRs cannot access the miRNA machinery in insect cells during this coordinated sequence of events. Alternatively, the imperfect duplex structure of miRNAs may preclude their binding by VSRs. In any case, the absence of effects of VSRs on miRNAs might represent an adaptation of insect hosts during evolution to limit the perturbation of gene expression upon viral infection. Of note, the VSR B2 was recently shown to suppress an inducible antiviral response in *Drosophila*, in addition to its role in suppressing RNAi [33]. It will be interesting to test if other VSRs can also block the upregulation of the antiviral gene *Vago* in infected flies.

The B2 and 1A proteins are encoded by FHV and DCV respectively, which belong to distinct families of (+) single stranded RNA viruses naturally infecting *Drosophila*. Both proteins inhibit the silencing of FHVΔB2 and sensitize flies to infection with DCV. Synergistic viral diseases in plants, namely the co-infection of a host plant with two unrelated viruses which elicits disease symptoms that are more severe than the sum of those induced in either single infection, have been shown to be dependent in some instances on VSRs [34]. Our data suggest that such viral synergism may also exist in insects and that VSRs could act as “synergism genes” becoming permissive agents for the development of infection.

The plant VSRs P19, P15 and P21 inhibit the antiviral silencing machinery of their host by binding to 21 nt siRNAs [35]. P19 also inhibits RNAi in mammalian cultured cells [12] and *in vitro* studies demonstrated that P19 and P21 proteins block siRNA-directed target RNA cleavage in *Drosophila* embryonic extracts by sequestering siRNA duplexes [17]. Here, we show that *in vivo* P19, P21 and P15 promote the accumulation of the FHVΔB2 replicon, sensitize flies to DCV infection and inhibit *ftz* silencing triggered either by long dsRNA or siRNA. Altogether, these data indicate that, in flies, these three VSRs mimic their function in plants by sequestering siRNAs. It is worthy to note that in a number of cases, plant viruses are transmitted by insect vectors [36] and may then have to face the antiviral RNAi response in distant organisms. Sequestering siRNAs, whose structure is conserved throughout evolution, may indeed represent an optimal strategy to adapt to otherwise evolutionarily divergent antiviral responses.

Although P0, P25, P38 and HcPro were characterized as potent VSRs in plants [37], they did not show activity in flies in any of our assays. We cannot exclude that this lack of activity results from imperfect biosynthesis of the viral suppressors in flies. Nevertheless, it is unlikely due to a deleterious effect of the Flag epitope as non-Flagged versions of these suppressors gave identical results (data not shown). P0 is an F-box like protein that induces the ubiquitinylation and the degradation of plant Argonautes [38]. Although the mechanisms by which P25 and P38 inhibit RNA silencing remain unclear, these two proteins do not appear to act by sequestering siRNAs [21]. It is therefore likely that the lack of activity of P0, P25 and P38 in flies reflects interactions of these VSR with host plant proteins whose structure and/or function are not conserved in flies. The HcPro protein from *Tobacco etch virus* (TEV) was shown to inhibit dsRNA-induced silencing in *Drosophila* S2 cells [39] and sequester siRNA from embryonic extracts, although less efficiently than P19 [17]. The lack of activity of HcPro from the ZYMV that we observed in our study may be due a divergence of sequence between HcPro proteins from TEV and ZYMV.

B2, 1A, P19, P15 and P21 VSRs that inhibit silencing induced by exogenously supplied dsRNAs were also capable of inhibiting the silencing mediated by siRNAs produced from an inverse repeat transgene. In addition, the insect VSRs B2 and 1A, and the plant VSR P19 inhibited the silencing of the *Drosophila* 297 and ZAM retrotransposons in heads and ovaries of adult flies, indicating that these suppressors interfere with the endo-siRNA pathway in flies [4], [6]. Their modest effect in gonads also suggests that they do not interfere with the piRNA-mediated silencing of TEs in this tissue.

The DCV-1A protein specifically binds long dsRNAs and does not interfere with RNAi when directly triggered by siRNAs [8]. Immunoprecipitation experiments showed that P19 and to a lesser extent B2 bind to TE-derived endo-siRNA in S2 cultured cell; additionally, B2 was specifically able to bind longer RNA species (Fagegaltier et al, in preparation). Together, these data suggest that the effects of VSRs on TE silencing is achieved through sequestration of TE-derived endo-siRNAs by P19, of long double-stranded TE RNA precursors by 1A and of both endo-siRNAs and their double-stranded precursors by B2.

It is remarkable that plants as well as insect VSRs can, besides their well-known roles in viral counterdefense mechanisms, consistently interfere with endo-siRNA pathway. Whether viruses can manipulate this pathway to improve their interaction with the host during the course of a natural infection and what the physiological implications would be are open questions.

## Materials and Methods

### Suppressor transgenic constructs

VSR cDNAs were first PCR amplified using the following templates : pBWDi-FHV-B2 kindly provided by C. Vaury (B2, Swiss-Prot P68831); pAC-DCV1A (1A, [8]; pBIN-P15, -P19, -P21, -P25 and -P38 constructs kindly provided by O. Voinnet (P15, P19, P21, P25 and P38, [21]; *Zucchini yellows mosaic virus* HcPro cDNA kindly provided by C. Desbiez (HcPro, Swiss-Prot P18479); pGBKT7-P0^CAB^ (P0, [38]. PCR primers were as follows: B2-F, 5′-CACCATGCCAAGCAAAC-3′; B2-R, 5′-CAGTTTTGCGGGTGG-3′; 1A-F, 5′-CACCATGGAATCTGATAAAAGTAT-3′; 1A-R, 5′-CTTGTCATCGTCATCCTTGTAAT-3′; P19-F, 5′-CACCATGGAACGAGC-3′; P19-R, 5′-CTCGCTTTCTTTTTCG-3′; P15-F, 5′-CACCATGCCTAAGTCG-3′; P15-R, 5′-CAGTTTAGAACGAAG-3′; P21-F 5′-CACCATGAAGTTTTTCTTTAATGA-3′; P21-R 5′-TACAGCTATACCGAGGATTT-3′; P25-F, 5′-CACCATGGATATTCTC-3′; P25-R, 5′-TGGCCCTGCGCGGAC-3′; P38-F, 5′-CACCATGGAAAATGATC-3′; P38-R, 5′-AATTCTGAGTGCTTGC-3′; HcPro-F, 5′-CACCATGTCGTCACAAC-3′; HcPro-R, 5′-CACCATGTCGTCACAAC-3′; P0-F, 5′-ATGGAAATTGAGTCTGTCAAGC-3′; P0-R, 5′-GCGTTGTAGCTCCTTTTG-3′

VSR cDNAs were cloned into the pENTR-D-TOPO vector (Invitrogen) and were subsequently recombined into pUASp vectors [24] adapted for the Gateway system (Drosophila Gateway® Vector collection, Carnegie Institution) to give C-terminal Flag tagged pPWF-VSR and non tagged pPW-VSR transgenesis vectors. VSR sequences in these constructs were verified by sequencing before establishing UAS>VSR transgenic stocks by standard injection procedure. For each VSR construct, three independent transgenic lines were tested in western blot assay as well as in *GMR*>IR[w] desilencing test. One highly expressed transgene insertion for each suppressor (Fig. S1) was chosen for further analysis.

### Transgenic and mutant stocks

The FHV RNA1 and RNA1ΔB2 flies are described in [7]. We obtained the *GMR*>IR[w] transgenic line from R. Carthew [28], the Ago2*^414^* mutant stock from H. Siomi [5], the *engrailed*>GAL4, *Tubulin*>GFP and *Tubulin*>GFP-ban transgenic stocks from S. Cohen [40], the *GMR*>GAL4, *da*>GAL4, *hsp*>GAL4 and UAS>GFP lines from the Bloomington Stock Center and the *Act5C*>GAL4 17a driver line (n°U192) from the Fly stocks of National Institute for Genetics from Japan (http://www.shigen.nig.ac.jp/fly/download/nigflyfiles/mutant.txt).

### Genetic crosses

To study the effect of the VSRs on the replication of FHV RNA1ΔB2, we genetically recombined the *da*>GAL4 driver with the UAS>VSR transgenic insertions. The resulting UAS>VSR; *da*>GAL4 stocks were then crossed to the UAS>RNA1ΔB2 transgenic line. For control, the UAS>RNA1ΔB2 line was crossed to the *da*>GAL4 driver line.

To monitor fly sensitivity to infection by DCV, the UAS>VSRs transgenic lines were crossed to the *Act5C*>GAL4 or *hsp*>GAL4 driver lines.

To analyze the effect of the suppressors on *white* silencing, we constructed an homozygous *w^1118^*, *GMR*>IR[w]; *GMR*>GAL4 stock by genetic recombination and crossed it to the homozygous UAS>GFP and UAS>VSR stocks. For controls, the UAS>GFP line, as well as all the UAS>VSR transgenic stocks were crossed to the homozygous *w^1118^*; *GMR*>GAL4 stock.

To analyze the effect of suppressors of bantam silencing, we recombined the *Tubulin*>GFP-ban sensor transgene with the *engrailed*>GAL4 driver on the third chromosome and crossed the resulting homozygous stock to the UAS>VSR lines.

### Quantitative RT PCR

To measure the steady state level of the FHV RNA1ΔB2, ten 6-days old female flies were collected from the progeny of each UASp>VSR; *da*>GAL4 stock crossed to homozygous UAS>RNA1ΔB2 flies. Same aged UAS>RNA1; *da*>GAL4 or UAS>RNA1ΔB2; *da*>GAL4 heterozygous flies were collected as controls. Total RNA extracts were prepared with Trizol Reagent (Invitrogen) according to the manufacturer's instructions, treated with DNAse I then reverse transcribed with iScript cDNA synthesis kit (Biorad). FHV RNA1 levels were then analyzed by real-time PCR on an Opticon2 Instrument (Biorad) using the Faststart Sybr-Green Mix (Roche) and the 5′ACCTCGATGGCAGGGTTT 3′ and 5′CTTGAACCATGGCCTTTTG 3′ primers. Template concentrations were normalized to endogenous reference *RpL23* and to the heterozygous UAS>RNA1ΔB2; *da*>GAL4 control calibrator using the ΔΔC_T_ method.

297 and ZAM transposon expression levels were measured in double heterozygous UASp>VSR; *da*>GAL4 and control heterozygous *da*>GAL4 2-days old females. Fifty heads and ovaries were separated manually and processed for total RNA extraction and reverse transcription as described above. FHV RNA1 levels were then analyzed by real-time PCR using the 297F 5′-AAAGGGCGCTCATACAAATG-3′, 297R 5′-TGTGCACATAAAATGGTTCG-3′ [30], ZAM-F 5′-ACTTGACCTGGATACACTCACAAC-3′ and ZAM-R 5′-GAGTATTACGGCGACTAGGGATAC-3′ [31] primers. Template concentrations were normalized to endogenous reference *RpL23* and to the heterozygous *da*>GAL4 control calibrator using the ΔΔC_T_ method.

In both experiments, RpL23 levels were measured using the RpL-F, 5′-CGGATCGATATGCTAAGCTGT-3′ and RpL-R, 5′-GCGCTTGTTCGATCCGTA-3′ primer pair.

### Viral infection

4–6 days old double heterozygous *Act5C*>GAL4; UAS>VSR or heat-shocked *hsp*>GAL4; UAS>VSR and control heterozygous *Act5C*>GAL4 or *hsp*>GAL4 flies were used in infection experiments. Viral stocks were prepared in 10 mM Tris-HCl, pH 7.5. Infections were done as described in [41] by injection (Nanoject II apparatus; Drummond Scientific) of 4.6 nL of a viral suspension (DCV, 2×10^5^ LD50 (50% lethal dose)/mL) into the thorax of flies. Injection of the same volume of 10 mM Tris-HCl, pH 7.5, was used as a control. For heat-shock induction of transgene expression, flies were incubated for 20 min at 37°C, followed by 30 min at 18°C and another 20 min at 37°C. After the treatment, flies were allowed to recover for 6 h at 25°C before infection. All survival experiments were performed at 22°C.

### *ftz* dsRNA and siRNA injection in embryos

A region of the *fushi tarazu* (*ftz*) gene was amplified with the T7-*ftz*-F 5′-GAATTGTAATACGACTCACTATAGGGCCAACATGTATCACCCCCA-3′ and T7-*ftz*-R 5′-GAATTGTAATACGACTCACTATAGGGCTGGCAAAGTCGCCATTCT-3′ primers using a *w^1118^* fly genomic DNA template. The PCR product was then used for dsRNA synthesis, using MEGAScript® RNAi Kit (Ambion®) according to the manufacturer's instructions. 5′ phosphorylated siRNA (21 bp) with dTdT 3′overhangs corresponding to the *ftz* gene (siFTZ 5′-UGCCUACUAUCAGAACACC-3′) was purchased from Eurogentec.

Embryos were collected from UAS>VSR; *da*>GAL4 stocks over a 30 min period at 25°C, hand dechorionated, and attached to a coverslip coated with double-stick tape. Embryos were then desiccated at room temperature, covered in 700 halocarbon oil and injected with a 1 mg/ml solution of dsRNA or siRNA using Eppendorf's sterile Femtotips® needles. After 36 hr recovery, the surviving embryos were scored for the number of ventral denticle belts. Embryos with four, five, or six denticle belts were scored as *ftz*.

### Eye pigment dosage

Assays were performed on 3 days old virgin females. Five heads per genotype were manually collected and homogenized in 50 µl of a freshly prepared solution of acidified methanol (0.1% HCl). Pigment was extracted by rocking tubes for 36 hr at 4°C. Homogenates were then incubated at 50°C for 5 min, clarified by centrifugation and optical density of each sample was read at 480 nm. Three independent extractions were performed for each genotype and the mean values of the absorption per extraction were calculated.

### Western-Blot analysis

For protein analysis, equal amounts of proteins were extracted from heterozygous UAS>VSR; *da*>GAL4 L3 larvae, boiled in Laemmli buffer and loaded on 15% SDS-PAGE. After transfer onto nitrocellulose membrane, equal loading was verified by Ponceau staining. Membranes were blocked in 5% milk, 1× PBS, 0.1% Tween, and incubated overnight with mouse HRP coupled anti-Flag antibody (1∶10000, Sigma). Detection was performed using Chemiluminescent Substrate (Pierce).

## Supporting Information

Figure S1 Expression of VSRs in transgenic animals. Third larval instar protein extract were prepared from transgenic larvae double heterozygous for the da>GAL4 driver and the indicated UAS>VSR transgene and analyzed by western blot using an anti-Flag antibody. Ponceau staining (bottom) shows equal protein loading between lanes.
(0.98 MB EPS)

Figure S2 VSRs do not suppress silencing by the bantam miRNA. Confocal microscopy of wing imaginal discs expressing the engrailed-GAL4 driver in their posterior half compartment (P) and either a control Tubulin>GFP or a Tubulin>GFP-ban sensor with bantam miRNA target sites in 3′ UTR. The quadrant pattern of GFP silencing by bantam in the wing pouch is not affected by expression of the indicated UAS>VSR transgenes in imaginal disc posterior compartment, indicating no obvious interference with the miRNA pathway by either VSR.
(4.43 MB TIF)

Movie S1 B2 confocal set. Confocal slice sets of UAS>B2; engrailed-GAL4, Tubulin>GFP-ban wing imaginal discs shown in Fig. S2
(56.46 MB AVI)

Movie S2 P15 confocal set. Confocal slice set of UAS>P15; engrailed>GAL4, Tubulin>GFP-ban wing imaginal discs shown in Fig. S2
(34.75 MB AVI)
