# Supplementary figures and images for: Viral Suppressors of RNA Silencing Hinder Exogenous and Endogenous Small RNA Pathways in Drosophila
Source: PLoS One. 2009 Jun 10;4(6):e5866. doi: 10.1371/journal.pone.0005866 (PMC2689938; doi:10.1371/journal.pone.0005866)

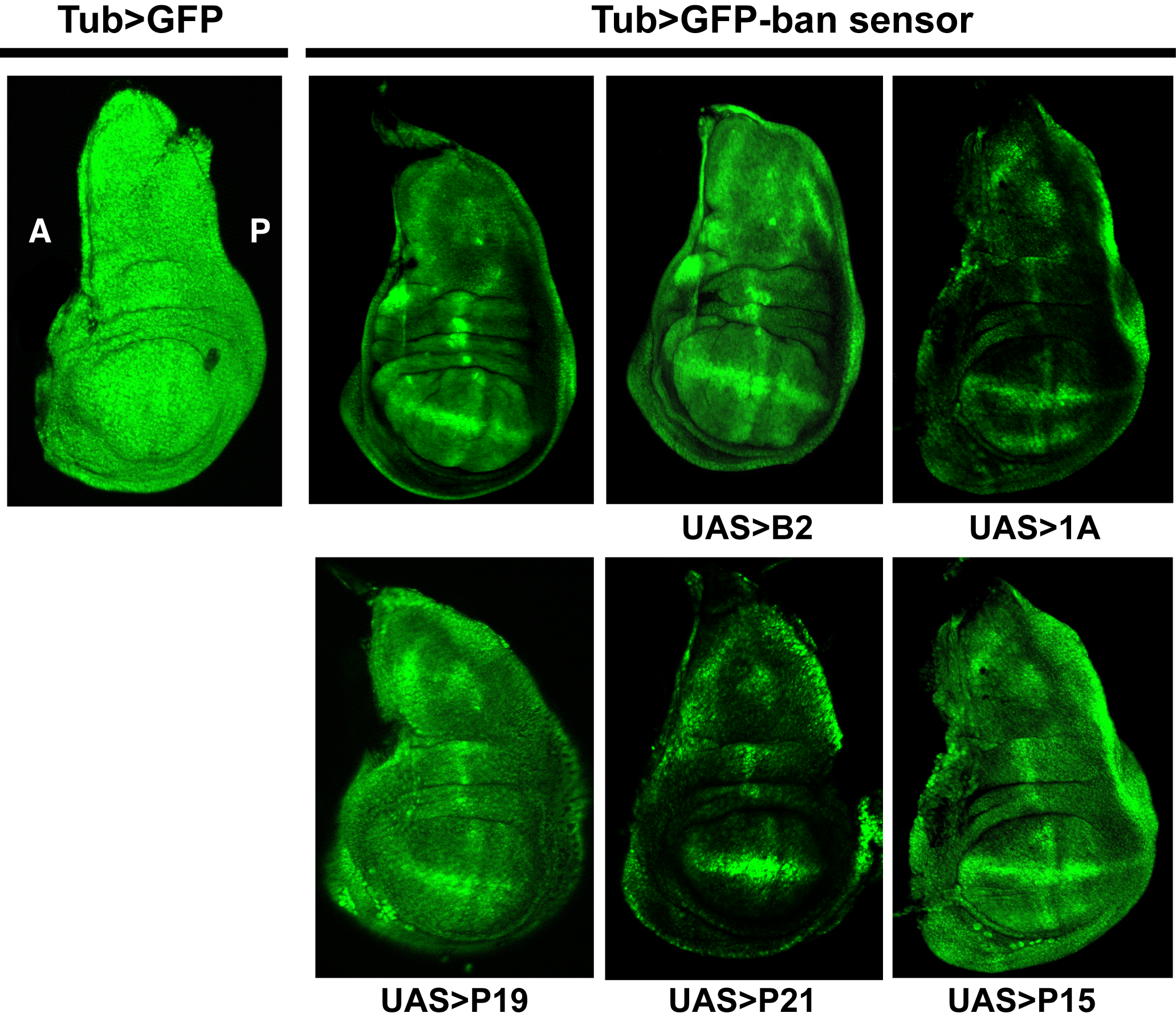

Supplement: Figure S2 — VSRs do not suppress silencing by the bantam miRNA. Confocal microscopy of wing imaginal discs expressing the engrailed-GAL4 driver in their posterior half compartment (P) and either a control Tubulin>GFP or a Tubulin>GFP-ban sensor with bantam miRNA target sites in 3′ UTR. The quadrant pattern of GFP silencing by bantam in the wing pouch is not affected by expression of the indicated UAS>VSR transgenes in imaginal disc posterior compartment, indicating no obvious interference with the miRNA pathway by either VSR. (4.43 MB TIF) [file pone.0005866.s002.tif]
